# Acute myocarditis mimicking ST-elevation myocardial infarction: A case report and review of the literature

**DOI:** 10.3892/etm.2015.2576

**Published:** 2015-06-12

**Authors:** TAO ZHANG, WEI MIAO, SHIXUAN WANG, MIN WEI, GUOHAI SU, ZHENHUA LI

**Affiliations:** 1 Department of Orthopedics, Jinan Central Hospital, Jinan, Shandong 250013, P.R. China; 2 Department of Cardiology, Jinan Central Hospital, Jinan, Shandong 250013, P.R. China; 3 Beijing University Medical School, Beijing 100191, P.R. China

**Keywords:** viral myocarditis, myocardial infarction, ST-elevation, coronary angiography

## Abstract

The present study describes the case of a young man aged 22 who had acute retrosternal pain, elevated cardiac markers and electrocardiographic ST-T changes, which led to an original misdiagnosis of acute myocardial infarction. The patient underwent immediate coronary angiography, which revealed normal coronary arteries. Finally, the diagnosis of viral myocarditis was made on consideration of his fever, scattered red dots on his arms and legs and other auxiliary examination results obtained in the following days, which were supportive of the diagnosis. The patient improved on antiviral and myocardial protection therapy and was discharged 2 weeks later. Viral myocarditis is a common disease with a variable natural history. It remains challenging for doctors to differentiate between acute myocarditis and myocardial infarction, particularly in the early stages. A diagnosis of myocarditis should be made on the basis of synthetic evaluation of the evidence, including medical history, clinical presentation and results of the available auxiliary tests, in order to provide guidelines for treatment.

## Introduction

Viral myocarditis is an inflammatory disease of the myocardium with heterogeneous clinical manifestations and progression, which make it challenging to diagnose and treat. It has been reported that myocarditis occurs in ~12% of young adults, and may contribute to other myocardial diseases, such as dilated cardiomyopathy and arrhythmogenic right ventricular cardiomyopathy (1). The clinical manifestations of myocarditis vary from mild disease with no symptoms, to heart failure and mortality. Myocarditis may be associated with heart tissue necrosis in some cases (2). There are numerous diagnostic methods for myocarditis, including cardiac magnetic resonance (CMR) imaging and endo-myocardial biopsy. CMR imaging is recommended as a credible and useful approach for monitoring the reversible and irreversible myocardial tissue injuries, and to distinguish acute myocarditis from healed myocarditis (3). Myocarditis may be caused by various viral infections, such as parvovirus B19, adenovirus and Coxsackie B virus. Therefore, virological detection of cardiac tissue is important for the diagnosis of myocarditis (4). Myocarditis may resemble myocardial infarction (5–7). In this study, a case of viral myocarditis in a patient with acute retrosternal pain, elevated cardiac markers and electrocardiographic ST-T changes similar to the clinical presentation of myocardial infarction is presented. Informed consent was obtained from the patient.

## Case report

### 

#### Primary diagnosis and treatment in the Emergency Room (ER)

A 22-year-old male was admitted to the ER with acute retrosternal pain and tightness that had persisted for >3 h. Upon presentation, on June 18, 2012, physical examination showed that his blood pressure was 120/70 mmHg, temperature was 37.2°C and heart rate was 63 bpm. First and second heart sounds were normal without any audible murmurs, rubs or gallops. No yellow discoloration of the skin or mucous membranes, bleeding or rashes were observed, and the remainder of the examination was normal. No history of hypertension or diabetes or family history of heart disease was reported, and the patient never smoked cigarettes or drank alcohol.

**Figure 1.**
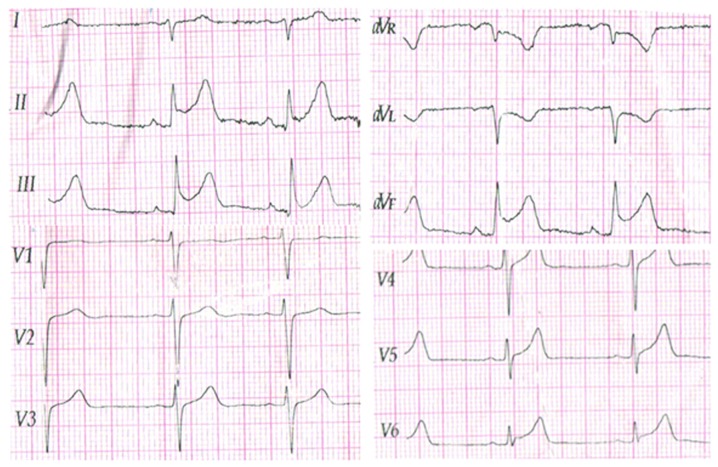
Twelve-lead surface electrocardiogram showing extensive ST-segment elevation in leads II, III and aVF (June 18, 2012).

A series of investigations were subsequently performed in the ER. The results of the cardiac enzyme tests disclosed that the troponin I (TnI) level was significantly elevated, up to 7.86 ng/ml (Table I). The stool studies were negative. Of note were the signs of ST-segment elevations in leads II, III and aVF on the electrocardiogram (ECG), suggestive of myocardial ischemia (Fig. 1). An echocardiogram showed that the patient's heart functioned normally with an ejection fraction of 63%, excluding the possibility of mitral valve prolapse syndrome.

**Figure 2.**
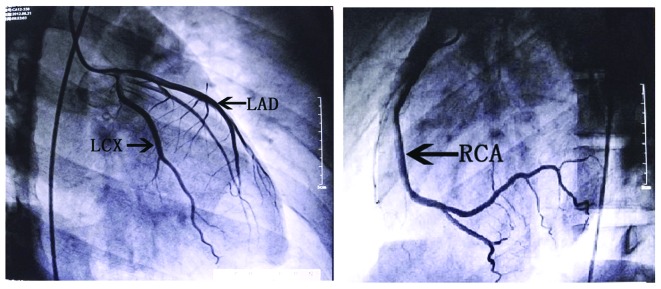
Coronary angiography revealing normal epicardial coronary arteries (June 18, 2012). LAD, left anterior descending coronary artery; LCX, left circumflex artery; RCA, right coronary artery.

Based on the findings of retrosternal pain, a typical ECG pattern of myocardial ischemia and an elevated TnI level, a tentative diagnosis of acute myocardial infarction was made. The patient immediately underwent coronary angiography, which showed normal epicardial coronary arteries (Fig. 2), contradictory to the diagnosis of myocardial infarction. On the second day of admission, June 19, 2012, he was transferred to the cardiology department for further diagnosis and treatment.

#### Clinical changes following admission

On the third day of admission, June 20, 2012, the temperature of the patient was 37.0°C in the morning but rose to 38.4°C at 7:00 p.m. In the following days, his temperature fluctuated between 35.5 and 38.4°C, and a range of necessary tests was performed to exclude other diseases that could also lead to body temperature rises (Table I). Scattered small red dots appeared on his feet, and the rash further expanded to his arms and legs 2 days later. A standard 12-lead ECG on the third day showed that the elevated ST segment in the II, III and aVF leads began to fall back, coupled with T-wave inversion. The TnI level increased to 8.470 ng/ml (Table I). Other examinations were performed during his hospitalization: A chest X-ray showed a normal heart size and mild markings in the lungs, with no clear indication of substantive lesion.

#### Secondary diagnosis, treatment and prognosis

**Figure 3.**
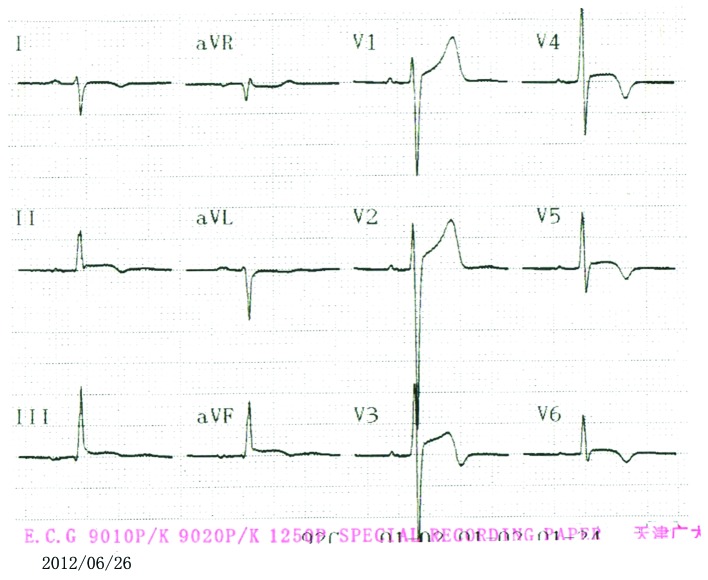
Review of the electrocardiogram on the ninth day demonstrated that the elevated ST segment in leads II, III and aVF had fallen back to the baseline level, coupled with T-wave inversion (June 26, 2012).

Evidence of a fluctuating fever and normal coronary arteries subverted the initial impression of acute myocardial infarction and aroused the suspicion of viral myocarditis. The diagnosis of myocarditis was confirmed by the clinical manifestation of a rash on the arms and legs. The patient was given an antiviral drug (acyclovir; 0.4 g, 3 times/day) and drugs to improve cardiac metabolism (trimetazidine; 20 mg, 3 times/day). Following 2 days of treatment, on the ninth day of admission (June 26, 2012), an improvement was observed, as indicated by the elevated ST segment in leads II, III and aVF falling back to baseline with T-wave inversion (Fig. 3) and the TnI level decreasing to 0.295 ng/ml.

The patient was discharged 2 weeks later. A post-discharge 1-month follow-up visit showed that the patient was recovering well.

## Discussion

Viral myocarditis is a common disease with a variable natural history. In the present case, the patient had typical retrosternal pain, elevated cardiac markers and electrocardiographic ST-T changes on admission, and was initially diagnosed with acute myocardial infarction; however, emergency coronary angiography demonstrated normal coronary anatomy. In addition, in the week following admission the patient presented with a flu-like illness manifesting as slight chills and fever. His cardiac enzymes subsequently decreased and the ST-segment elevation gradually decreased. Other unique characteristics of myocarditis in this case were the temperature fluctuations and the rash on the patient's arms and legs, which appeared later during his hospitalization. Based on these clinical manifestations and auxiliary examination, the diagnosis of acute myocarditis could be confirmed.

It is likely that the typical clinical presentations of myocardial infarction, such as chest pain, ST-segment elevation and incremental serum markers, appear in patients diagnosed with myocarditis (8). The diagnosis of myocarditis is often empirical. Physicians should take into consideration several lines of evidence, such as clinical presentation, ECG alterations and cardiac enzyme changes, prior to making a diagnosis. In addition, epicardial coronary artery disease should be excluded. The following discussion aims to provide a comprehensive description of viral myocarditis, including the pathogenesis, clinical presentation, development of diagnostic methods and treatment.

With regard to the etiology of the condition, the advancement of molecular biology has led to the identification of a number of different viruses and virus subtypes that are causative factors for myocarditis. The more common viruses are coxsackievirus, adenovirus, cytomegalovirus and parvovirus B19, as well as hepatitis C, influenza, herpes simplex and Epstein-Barr viruses (9,10).

In the first phase of myocarditis, the virus enters and proliferates in the myocardium, causing direct myocardial damage, followed by the initiation of the innate immune response. Both the direct myocardial damage caused by the virus and the subsequent immune process result in destruction of the cardiomyocytes and lead to elevations in the serum cardiac Tn and enzyme levels, mainly to eliminate as many virus-infected cells as possible to control the infection, which includes the activation of complement (a process that produces both cell lysis and substances chemotactic for neutrophils and macrophages), the activation of various cytokines and the infiltration of T lymphocytes and macrophages, which can be detected by biopsy (9,11). Following the first phase, patients will recover or progress into the second phase in which the adaptive immune response is activated.

Molecular mimicry accounts for part of the persisting myocardial damage in the second phase, as the virus antigens and the myocardial cells share similar epitopes, which activate the B cells to produce cross-reacting antibodies and thus activate the effector T cells (12–14). Lawson *et al* (14) induced persisting myocarditis in the susceptible BALB/c strain of mice with mouse cytomegalovirus, and found that autoantibodies to cardiac myosin were produced following mouse cytomegalovirus infection. These affinity-purified anti-cardiac myosin antibodies cross-reacting with mouse cytomegalovirus proteins suggest that viral infection may modulate the immune recognition of the common epitopes shared between the mouse cytomegalovirus proteins and the heavy chain of myosin (14). Cross-reacting antibodies with auto-antigens have also been found in patients with myocarditis (15). In the third phase, the intensity of the immune response is downregulated and fibrosis starts (16,17). As a result, the persistent low-grade immune response leads to extensive myocardial injury and, eventually, dilated cardiomyopathy (17,18).

The clinical manifestations of viral myocarditis are highly variable, ranging from asymptomatic to acute heart failure. Acute myocarditis often presents with a flu-like illness, including fever, myalgia, malaise, nausea and vomiting, for a few days to 3 weeks before any cardiac symptoms appear (19). The majority of patients will make a full recovery; however, a number of patients can rapidly progress to chest pain, respiratory distress, arrhythmia or even heart failure, which necessitates hospital admission. Further physical examination may reveal cardiac pathological signs, such as sinus tachycardia, low first heart sounds, gallops and murmurs of mitral or tricuspid insufficiency, which are not specific for myocarditis. Other unspecific signs, such as the appearance of skin rashes, can also be found in certain patients (20). In the current case of viral myocarditis, the patient had fever with temperature fluctuations between 35.5 and 38.4°C and subsequently showed a rash on the arms and legs in the week following admission.

Several diagnosis modalities can be helpful in the diagnosis of myocarditis, including electrocardiography and cardiac biomarkers. Damage of the myocytes causes abnormal electrical activity of the heart, which leads to abnormalities in the ECG, including ST-T wave changes, ST elevation, atrial and ventricular arrhythmias, atrial-ventricular and intraventricular conduction defects and variant early repolarization (21); however, these ECG alterations are non-specific. Myocarditis may share similar ECG changes with myocardial infarction. TnT, TnI and creatine kinase (CK)-MB are the most commonly used cardiac biomarkers. Cardiac Tn is mainly elevated in the acute phase of myocarditis and decreases gradually as the patient improves (22). The sensitivity of cardiac biomarkers to myocardial injury varies. As with electrocardiography, cardiac Tns are non-specific for myocarditis, although they are more sensitive than CK levels. Consistently, in this case of viral myocarditis, the TnI level began rising from the first day of admission, peaked at 8.470 ng/ml on the third day but then slumped and reached 0.295 ng/ml on the ninth day. ST-segment elevations in the II, III and aVF leads on the ECG, accompanied by acute retrosternal pain and elevated cardiac markers, led to the initial incorrect diagnosis of myocardial infarction.

Since only certain patients present with elevated cardiac enzymes, the reliability of cardiac enzymes for diagnosing myocarditis remains uncertain and should be investigated further. In addition to cardiac Tns, the level of brain natriuretic peptide (BNP) measured in the plasma may be a useful biochemical marker for myocarditis, and high concentrations of BNP may correlate with poor prognosis in patients with myocarditis (23). Caforio *et al* (22) suggested that the log-BNP concentration could be a quantitative biochemical marker of myocarditis in Kawasaki disease. Viral culture should be considered to help identify the virus responsible for the disease, although the virus can only be isolated from the blood in a minority of cases. Polymerase chain reactions, immunoglobulin antibody assays and viral titers can help to improve the possibility of detecting the pathogen.

Echocardiography can also be of use in the diagnosis of myocarditis. The echocardiographic findings suggestive of myocarditis are left ventricular dilation, decreased function, systolic and diastolic dysfunction and regional wall motion abnormalities. Furthermore, patients may have myocardial interstitial edema, which can also be detected by echocardiography through the thickness of the ventricular wall (24). There are additionally non-specific echocardiographic characteristics associated with acute myocarditis. In the present study, normal heart function was suggested by an echocardiogram. Developments in technology have brought new progress in diagnosis. Notably, recent reports have recommended that speckle-tracking echocardiography, characterized by the precise evaluation of regional contractility, should be used as an adjunctive tool for the diagnosis of acute myocarditis and inflammatory cardiomyopathy (25,26).

In recent years, cardiac magnetic resonance imaging (CMRI) has emerged as one of the most useful imaging devices for detecting and diagnosing myocarditis, as it can provide various means to visualize and quantify myocardial inflammatory changes (27,28). CMRI, however, is not accepted by a proportion of patients with suspected myocarditis due to the considerable expense. The current patient was one such case.

Finally, the histological and immunological evaluation of biopsies can be used in the diagnosis of acute myocarditis. Endomyocardial biopsy (EMB) is not a routine diagnostic method in the majority of cases of suspected acute myocarditis, since it is an invasive approach and has a probability of sampling error due to the characteristic patchy inflammation and variability in observer interpretation. A 2013 position statement from the European Society of Cardiology Working Group on Myocardial and Pericardial Diseases (22) recommended heart biopsy as a routine test for all cases of suspected myocarditis. Conversely, the routine application of EMB was not recommended by the 2013 American College of Cardiology Foundation/American Heart Association (29); therefore, no consensus has been reached with regard to the application of EMB in the diagnosis of myocarditis. EMB was not performed in the present case.

Advances in histological and molecular genetic technology, such as the polymerase chain reaction and *in situ* hybridization, have improved the efficiency of identifying viral genomes and cardiac inflammation. According to the Canadian Cardiovascular Society Consensus Conference guidelines on heart failure (updated 2009), EMB evaluation for myocarditis should include the use of histopathological markers of inflammation and necrosis, immunohistochemical markers and the assessment of viral particles (30); however, it has been indicated that the histological diagnosis of myocarditis based on the Dallas criteria lacks sensitivity and specificity (31). Additionally, an absence of sensitive markers for an active immunological process can limit the use of histopathological analysis (32). Immunohistochemical techniques can enable the quantification and identification of activated inflammatory cells, including T lymphocytes, B cells, macrophages and natural killer cells. Among those cells, T lymphocytes are essential for diagnosing active myocarditis. Immunostains for cell-specific markers may also help confirm the presence of myocardial inflammation. The analysis of viral replication in the myocardium through the use of the polymerase chain reaction and *in situ* hybridization can help quantify the specific viral variants accounting for myocardial damage. Previous findings using these novel diagnostic tests point toward a broader spectrum of viral genomes responsible for acute myocarditis, indicating a shift from enterovirus and adenovirus to parvovirus B19 and human herpes-virus 6 as the viruses most frequently causing acute myocarditis (33–35).

With regard to the treatment of the condition, ~50% of the patients with acute myocarditis are likely to spontaneously recover within a month, ~25% will develop persistent impaired cardiac function and up to 25–30% may either progress to dilated cardiomyopathy, making heart transplantation a necessity, or succumb to the condition (35,36).

For symptomatic treatment of acute myocarditis, physical activity should be avoided, as sports may promote viral replication and increase the burden on the heart. As long as the patient has symptoms such as chest pain, respiratory distress, ECG abnormalities, increased levels of TnI/T or CK-MB, symptomatic treatment should be undertaken, including diuretics, β-blockers, angiotensin-converting enzyme-inhibitors or angiotensin II receptor blockers. Patients presenting with heart failure should be administered suitable drugs, including positive inotropic agents, vasodilators and diuretics. In the case of severe heart failure, mechanical circulatory support should be introduced, such as an intra-aortic balloon pump or a left ventricular assist device (33,37). Heart transplantation should be considered if the aforementioned measures fail. Arrhythmia is common, particularly ventricular arrhythmia. If patients present with severe refractory ventricular arrhythmias or atrioventricular blocks, they may require antiarrhythmic medication or the insertion of implantable cardioverter defibrillators or temporary pacemakers, respectively (32).

Since patients are generally diagnosed with myocarditis within days or weeks after the initial viral infection, antiviral therapy is seldom used in clinical practice in the early phase of myocarditis, although antiviral therapy has been reported to have a positive effect in the acute viremic stage (38). A patient with parvovirus-B19-associated fulminant myocarditis was reported to show a complete recovery with immunosuppressive and antiviral therapy (intravenous immunoglobulin and acyclovir) within 2 weeks (39). Furthermore, the antiviral effect of interferon-β therapy, enhanced by the transcription suppressor 4E-BP1, has been reported in myocarditis induced by coxsackievirus B3 (40). Consistently, in the present study, the patient was administered the antiviral drug acyclovir and cardiac metabolism-promoting drugs and recovered within 2 weeks.

Immune suppression may be beneficial in patients with systemic disease-related or autoimmune myocarditis but may increase virus replication and worsen myocardial injury in viral myocarditis. A recent study showed that immunosuppressive treatment taken immediately on detection of sclerotic heart disease proved effective in preventing cardiac damage progression (41). Similarly, an improved performance of immunosuppressive therapy (azathioprine and prednisone) was reported for children with chronic myocarditis, regardless of the presence of viral infection, compared with conventional therapy (42). By contrast, Hia *et al* (43) reviewed the impact of immunosuppressive therapy on the outcome of acute myocarditis in children, and the 18 years of data suggested that immunosuppressive therapy does not significantly improve outcomes in children with acute myocarditis, providing negative evidence for its routine use (43). It is therefore necessary to evaluate the type of myocarditis carefully prior to starting the immunosuppressive therapy in order to avoid its ill effects.

Despite considerable progress, it remains a daunting challenge for physicians to discriminate between acute myocarditis and myocardial infarction, particularly in the early phase. An integrated assessment and evaluation of evidence, including medical histories, clinical presentation and results of other auxiliary tests, are necessary for the accurate diagnosis of myocarditis and can guide treatment accordingly. In the current case, a patient with viral myocarditis presented with retrosternal pain, elevated cardiac marker levels and ST-T changes on the ECG, similar to the clinical manifestations of acute myocardial infarction. Clinical manifestations, including a fever with temperature fluctuations and the appearance of a rash on the arms and legs, coronary angiography and the results of auxiliary examinations, could aid in the differential diagnosis between acute myocarditis and myocardial infarction. The etiology, pathology, diagnostics and therapy of myocarditis remain controversial. Future investigations are required to further unravel these questions.

## Figures and Tables

**Table I.** Results of the laboratory tests.

Test	Value	Reference
WBCs (x10^9^/l)	5.75	4–10
Neutrophils (%)	55.1	50–70
Hemoglobin (g/l)	149	110–160
Triglyceride (mmol/l)	1.06	0.30–1.80
Total cholesterol (mmol/l)	3.45	3.40–6.50
CK-MB (ng/ml)	31.4	0–3.7
MYO1 (ng/ml)	59.5	0–73
Troponin I (ng/ml)		0.00–0.090
June 18, 2012	7.860	
June 20, 2012	8.470	
June 26, 2012	0.295	
BNP (pg/ml)	15.2	0.0–100.0
ESR (mm/h)	11	2–20
ASO (IU/ml)	62	0–200
U&E/LFT	Normal	
RF	Negative	
PPD	Negative	

WBCs, white blood cells; CK-MB, creatine kinase isoenzyme; MYO1, myohemoglobin; BNP, brain natriuretic peptide; ESR, erythrocyte sedimentation rate; ASO, anti-streptolysin O; U&E, urea, creatinine and electrolytes; LFT, liver function test; RF, rheumatoid factor; PPD, purified protein derivative.
